# A Case of Non-purpuric Neonatal Alloimmune Thrombocytopenia Secondary to ABO Incompatibility

**DOI:** 10.7759/cureus.38728

**Published:** 2023-05-08

**Authors:** Kylee Arthurs, Barbara S Suening, Ana Paraiso, Anjali Patel, Karla Diaz Ayllon

**Affiliations:** 1 Medical School, Edward Via College of Osteopathic Medicine, Auburn, USA; 2 Medical School, Edward Via College of Osteopathic Medicine, Spartanburg, USA; 3 Neonatology, HCA Orange Park Medical Center, Orange Park, USA

**Keywords:** abo compatibility, igg antibody, fitzpatrick type v, nonpurpuric, neonatal alloimmune thrombocytopenia

## Abstract

Neonatal alloimmune thrombocytopenia (NAIT) is a condition in which maternal IgG antibodies are directed against fetal platelets and cross the placenta, destroying fetal thrombocytes. It is typically caused by maternal alloimmunization to human leukocyte antigens (HLA). ABO incompatibility, on the other hand, is a rare cause of NAIT due to the variable expression of ABO antigens on platelets. Here, we present the case of a first-time mother (O+) who delivered a 37-week 0-day gestation newborn (B+) that was anemic and jaundiced with critically high total bilirubin levels. This required the initiation of phototherapy and intravenous immunoglobulins. Despite treatment, jaundice was slow to improve. Given infectious concerns, a complete white blood cell count was ordered. Incidentally, it revealed severe thrombocytopenia. Platelet transfusions were administered, although only minimal improvement was observed. This warranted maternal testing for antibodies to HLA-Ia/IIa, HLA-IIb/IIIa, and HLA-Ib/IX antigens given suspected NAIT. Results returned negative. Due to the severity of the condition, patient care was continued at a tertiary facility. When screening for NAIT, special consideration should be given to type O mothers with ABO incompatibility to their fetus - they can uniquely make IgG against A or B antigens, which, unlike IgM and IgA, can cross the placenta and cause potential sequelae harming the newborn. Early recognition and timely management of NAIT are important to prevent certain complications, such as fatal intracranial hemorrhage and developmental delay.

## Introduction

Neonatal alloimmune thrombocytopenia (NAIT) is a condition in which maternal IgG antibodies directed against fetal platelet antigens cross the placenta, resulting in the destruction of thrombocytes. Clinical manifestations in the newborn vary widely, although they typically present as a petechial rash or forms of spontaneous bleeding. In severe cases, intracranial hemorrhaging may occur [1]. Thrombocytopenia is defined as platelet levels below 150,000/uL, with severe thrombocytopenia being less than 50,000/uL [1]. Pathogenesis is typically due to HLA-Ia incompatibility between the mother and fetus. This can occur when a newborn’s father is HLA-Ia positive, but the mother is not. Maternal anti-HLA-Ia antibodies cross the placenta reaching the fetus, causing a Coombs positive reaction [2]. Other causes of incompatibility include HLA-Vb in the Caucasian population and HLA-IV in the Asian population [2]. Rarely, it may be due to a mutation resulting in the failure of CD36 expression, which is a scavenger receptor protein found in platelet cells, RBCs, and endothelial cells [3]. This mutation is found in 5% of the Asian and African population [3].

Interestingly, platelets express small amounts of A and B antigens on their surface [4]. In an O+ mother and AB incompatible fetus, IgG antibodies crossing the placenta may lyse both RBCs and platelets. This is not a common finding because the expression of ABO antigens on platelets is highly variable and is only strongly expressed in 4-7% of individuals [5].

There are limited treatment options for NAIT when diagnosed before delivery. Intravenous immunoglobulins (IVIG) and steroids are used as a means to increase platelet count prior to labor [6]. After delivery, IVIG can be given to the neonate in an attempt to neutralize the maternal antibodies [6]. Platelet transfusions may also be utilized. Current treatment protocols suggest platelet transfusions when bleeding is present and platelets are less than 50,000/uL and also suggest prophylactically transfuse at less than 20,000/uL [7].

Overall, NAIT is relatively uncommon, occurring in one in 1,000 live births. In addition to being at increased risk for excessive bleeding and intracranial hemorrhaging, NAIT may have lasting neurologic complications causing developmental delay [8]. Early recognition and timely management of NAIT are therefore important to prevent these complications and to provide the best possible outcome.

## Case presentation

**Figure 1:**
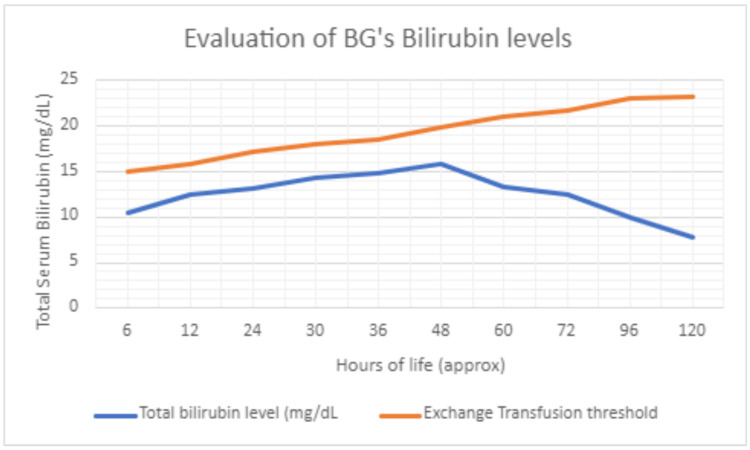
Evaluation of BG’s bilirubin levels BG, baby girl

A 28-year-old G1P0 African American female with a past medical history of anemia presented to labor and delivery in active labor. She was positive for group B streptococcus but otherwise had an uncomplicated pregnancy. She delivered a baby girl (BG) at 37 weeks 0 days gestation. BG was 5 lbs 8.9 oz with an APGAR score of 8 and 9 at 1 and 5 minutes, respectively. Physical exam revealed a well-appearing term newborn with well-perfused skin and no evidence of petechiae. Cord blood testing unveiled that BG was blood type B+ and Coombs positive with a direct antiglobulin test (DAT) of 2+. Due to bloodwork at six hours of age showing a total bilirubin level of 10.5 mg/dL and a direct bilirubin of 0.6 mg/dL, the infant was transferred to the neonatal intensive care unit (NICU), where she was started on triple UV phototherapy. Repeat blood work was ordered for 12 hours of age and was trended throughout the admission (Figure 1). Prior to the repeat blood draw, BG demonstrated thermoregulation issues that warranted a full CBC. Ampicillin and gentamicin were administered for sepsis prophylaxis. The results of the blood work at 12 hours of age revealed an up-trending total bilirubin to 12.4 mg/dL, white blood cell count (WBC) of 27.4 × 103/uL, and platelet count of 18 × 103/uL with no left shift in WBCs (Table 1).

**Table 1:** Trending BG’s platelets, total serum bilirubin, and white blood cell count BG, baby girl

Day of life	Platelet count (x10^3^/uL)	Total serum bilirubin (mg/dL)	White blood cell count (x10^3^/uL)
1	PM: 18, s/p transfusion: 26	AM: 10.5, PM: 12.4	PM: 27.7, s/p transfusion: 33.4
2	AM: 28, PM: 34	AM: 13.1, PM: 14.9	AM: 31.3, PM: 24.3
3	AM: 28, PM: 19, s/p transfusion: 73	AM: 15.8, PM: 13.3	AM: 21.2
4	AM: 45, PM: 7, s/p transfusion: 14	PM: 11.1	AM: 16.2
5	AM: 19	AM: 9.9	
6	AM: 14	AM: 7.8	

Figure 1 follows the bilirubin levels of BG from six hours of life until discharge to a tertiary care center. Her phototherapy was ceased on her fifth day of life due to a continued decrease in bilirubin and the increased gap with the threshold for exchange transfusion [9].

In addition to the triple UV phototherapy during BG’s NICU stay, IVIG was administered and platelets were trended. An ultrasound of her head ruled out intracranial hemorrhage. Upon identification of the newborn’s thrombocytopenia, blood from the mother was collected to evaluate for antibodies against platelets. The urine drug screen returned negative.

On day two of life, BG’s repeat platelet count was 27 × 103/uL after her first platelet transfusion. Another dose of IVIG and 10 mL/kg platelets were given. Her platelets rose only to 28 × 103/uL, but hematocrit was found to be 25%. Another platelet transfusion was ordered, as well as a blood transfusion of 15 mL/kg. Physical exam at this time continued to show jaundice but without visible petechiae or other abnormalities.

On day three of life, BG was still receiving triple UV phototherapy. Her repeat blood work continued to show an upward trend in total serum bilirubin and a downward trend in platelets (Table 1). Antibiotics were discontinued after a 48-hour sepsis rule-out. Given persistently low platelets, the decision was made to send a urine sample to rule out congenital cytomegalovirus, as this is known to also cause thrombocytopenia. The results later came back negative. Platelets were ordered again, with the follow-up platelet level being 73 × 103/uL. A process had been started to try to find HLA-Ia negative platelets for transfusion.

On day four of life, BG was still jaundiced and receiving triple light phototherapy. Given the improvement in bilirubin levels, she was weaned to double phototherapy. Her platelets in the morning were found to be 45 × 103/uL, with an order to transfuse platelets at less than 30 × 103/uL. She was still jaundiced. Later in the day, her platelets were read as 7 × 103/uL. However, lab personnel called to report that the sample was clotted. Based upon platelet trends throughout NICU admission, the decision was made to give another platelet transfusion. At that time, it was becoming difficult to obtain platelets - the NICU facility was unable to provide small aliquots needed for her size, which led to the wasting of large amounts of platelets.

**Figure 2:**
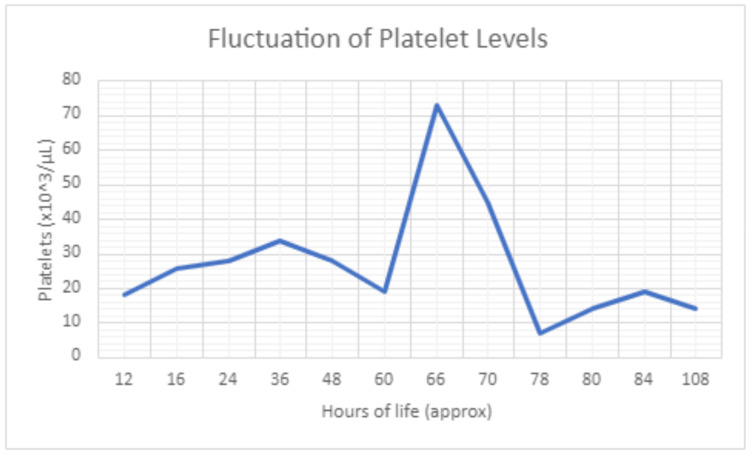
Fluctuation of platelet levels

On the morning of her fifth day of life, her platelets were found to be 14 × 103/uL and then rose to 19 × 103/uL. Her total bilirubin had stabilized and phototherapy had been discontinued. On her sixth day of life, her platelets were still critically low at 14 × 103/uL (Figure 2 and Table 1). The decision was then made to transfer her to a tertiary care center with a level three (highest acuity) NICU. At this facility, providers would have access to HLA-Ia antigen negative platelets and a system to allot platelets in the correct volume without having to waste a large amount of blood products. Maternal antibodies against HLA-Ia/IIa, HLA-IIb/IIIa, and HLA-Ib/IX antigens ordered upon initial suspicion of NAIT returned negative.

At 16 hours of life, BG received her first platelet transfusion, with a corresponding increase. At 60 hours of life, she received her second platelet transfusion raising her levels from 19K to 75K. At 78 hours of life, she had a value of 7K; however, the lab called to report clumping of blood samples. She received another platelet transfusion at this time.

## Discussion

Maternal alloantibodies to red blood cell antigens can lead to hemolytic disease of the fetus and newborn (HDFN), characterized by jaundice due to the buildup of bilirubin from hemolysis [10]. HDFN can result in neurological complications if the blood-brain barrier is breached [11]. RhD antigen incompatibility between mother and fetus is the most commonly discussed cause, but other antigens, including those determining ABO blood typing, may also cause HDFN [12]. Antibody production against non-self-antigens begins at three to six months of age. In particular, individuals with the type O blood group can uniquely produce IgG antibodies to A and B proteins, which can cross the placenta and trigger severe HDFN in the neonate [10].

BG's presentation required ruling out NAIT as a potential diagnosis, given the presence of ABO incompatibility identified through Coombs testing at birth. Jaundice and elevated bilirubin levels prompted the initiation of HDFN treatment protocols. It was not until hypothermia and thrombocytopenia were observed during a sepsis workup that the possibility of another underlying condition was considered. Despite transfusions, persistent thrombocytopenia persisted until NAIT was eventually diagnosed after ruling out CMV.

As mentioned above, the most common cause of NAIT in African and Caucasian ancestry is maternal-fetal incompatibility for HLA-Ia [3]. The mother, in this case, was tested for antibodies to HLA-Ia/IIa, HLA-IIb/IIIa, and HLA-Ib/IX antigens, which were negative, suggesting an alternative etiology for NAIT. It is possible that BG had an uncommon case where her platelets expressed high levels of AB antigens, although other HLA haplotypes were not tested for [7]. Additionally, mutations to glycoprotein IV/CD36 have been associated with NAIT in those of African and Asian descent [3].

In NAIT, petechiae followed by thrombocytopenia are typically the first presenting symptoms, yet our patient deviated from this norm. BG's Fitzpatrick type V complexion didn't reveal any apparent petechial rash, and yet her platelet count was remarkably low, resulting in speculation for potential oversight regarding petechiae.

This case introduced the importance of increased screening for NAIT. Based on previously published studies, trials of universal screening tools have been conducted, which focused on noninvasive HPA-1a typing. They have shown promise in reducing the associated risks of NAIT by 75% [8]. Despite promising results, the aforementioned tools have yet to be implemented in clinical settings, and preventive measures are not currently available [8]. It is also suggested that all ABO incompatible babies can start with a CBC to monitor platelet count. Neonates born to mothers with O+ blood who are found to have an ABO incompatibility, however, should be immediately screened for NAIT rather than focusing solely on bilirubin levels. It is also important for research to continue into the causes of NAIT and the best treatment options available. As with any treatment plan, the current use of IVIG, steroids, and transfusions all carry their own risks. In cases as severe as this, in which thrombocytopenia is persistent despite several transfusions, it is important to elucidate better treatment options. It should also be discussed that hospitals with NICU level II or higher should have the resources available to accurately aliquot thrombocytes for smaller quantities of transfusion. Although others may argue that preparing platelets for the possible occurrence of NAIT in a level II is impractical due to the low occurrence, it should still be considered to decrease the waste of very precious resources.

## Conclusions

Screening for NAIT, particularly in type O mothers with ABO incompatibility, is suggested because they can uniquely make IgG against A or B antigens. As IgG antibodies can cross the placenta, unlike IgM and IgA, they can cause complications in the newborn by causing the destruction of cells expressing AB antigens (i.e., RBC and thrombocytes). Early recognition and timely management of NAIT is important to prevent serious complications, including but not limited to, excessive bleeding, fatal intracranial hemorrhage, and developmental delay. Further research should be done into the different causes of NAIT as well as improvement in the treatments available.
